# Endothelial Microvesicles Induce Pulmonary Vascular Leakage and Lung Injury During Sepsis

**DOI:** 10.3389/fcell.2020.00643

**Published:** 2020-07-16

**Authors:** Danyang Zheng, Jie Zhang, Zisen Zhang, Lei Kuang, Yu Zhu, Yue Wu, Mingying Xue, Hongliang Zhao, Chenyang Duan, Liangming Liu, Tao Li

**Affiliations:** State Key Laboratory of Trauma, Burns and Combined Injury, Shock and Transfusion Department, Research Institute of Surgery, Daping Hospital, Army Medical University, Chongqing, China

**Keywords:** endothelial microvesicles, vascular leakage, microRNA-23b, lung injury, sepsis

## Abstract

Sepsis is a prevalent severe syndrome in clinic. Vascular leakage and lung injury are important pathophysiological processes during sepsis, but the mechanism remains obscure. Microvesicles (MVs) play an essential role in many diseases, while whether MVs participate in vascular leakage and lung injury during sepsis is unknown. Using cecal ligation and puncture induced sepsis rats and lipopolysaccharide stimulated vascular endothelial cells (VECs), the role and the underlying mechanism of endothelial microvesicles (EMVs) in pulmonary vascular leakage and lung injury were observed. The role of MVs from sepsis patients was verified. The results showed that the concentration of MVs in blood was significantly increased after sepsis. MVs from sepsis rats and patients induced apparent pulmonary vascular leakage and lung injury, among which EMVs played the dominant role, in which miR-23b was the key inducing factor in vascular leakage. Furthermore, downregulation and upregulation of miR-23b in EMVs showed that miR-23b mainly targeted on ZO-1 to induce vascular leakage. MVs from sepsis patients induced pulmonary vascular leakage and lung injury in normal rats. Application of classic antidepressants amitriptyline reduced the secretion of EMVs, and alleviated vascular leakage and lung injury. The study suggests that EMVs play an important role in pulmonary vascular leakage and lung injury during sepsis by transferring functional miR-23b. Antagonizing the secretion of EMVs and the miR-23b might be a potential target for the treatment of severe sepsis.

## Introduction

Sepsis is a life-threatening organ dysfunction caused by a dysregulated host response to infection, which leads to tissue damage and organ dysfunction even multiple organ dysfunction syndrome (MODS) with high mortality (Rhodes et al., 2017). Recent epidemiologic studies showed that sepsis incidence rate has been up to 535 cases per 100,000 person-years and still rising in the United States, meanwhile the in-hospital mortality was up to 25−30%, which indicated that sepsis remains a huge task to be overcome all around the world (Fleischmann et al., 2016). The lung is usually one of the first failure organs during the development of multiple dysfunction after sepsis (Wu et al., 2007), and vascular leakage plays a fundamental role in lung injury, which is characterized by the endothelial barrier dysfunction and vascular endothelial permeability increment, meanwhile it is the crucial reason for the organ dysfunction in sepsis (Hou et al., 2017). Although the mechanism of vascular leakage has been studied for years, there is no effective treatment clinically, and it needs to be further investigated.

Plenty of cells in circulation are stimulated during sepsis, such as platelets, vascular endothelial cells and leukocytes, and may secrete microvesicles (MVs) subsequently (Becker et al., 2016; Karpman et al., 2017; Todorova et al., 2017). MVs are defined as membrane derived vesicles which are secreted into the extracellular environment by almost all types of cells under both normal and stress conditions such as diabetes and atherosclerosis (Tkach and Thery, 2016). The concentration of MVs, especially for platelet MVs (PMVs) and endothelial MVs (EMVs) in circulation, is positively correlated to the severity of diseases (Mastronardi et al., 2011; Hoefer et al., 2015; Martinez and Andriantsitohaina, 2017). Cheow et al. (2016) found that leukocyte MVs (LMVs) and EMVs could promote the generation of thrombin during myocardial infarction, and led to the disorder of coagulation and fibrinolysis system. Laher (2011) found that MVs could regulate the relaxation of blood vessel after sepsis by inducing the generation of superoxide anion and inducible Nitric Oxide Synthase (iNOS) (Lovren and Verma, 2013). Whether MVs play an important role in vascular leakage after sepsis remains unknown.

It is considered that MVs participate in the pathological processes of tissue and organ dysfunction either by directly interacting with cells or by transferring bioactive molecules as proteins, DNA, miRNA, lipids into target cells. Recent studies identified that miRNAs in MVs played important roles in some diseases (Zhang et al., 2010; Zhou et al., 2014; Xu et al., 2017; Zheng et al., 2017). For example, the MV-carrying miR-105 could reduce the expression of tight junction proteins such as ZO-1, which was related to the destruction of vascular barrier (Zhou et al., 2014). Hence, we hypothesized that MVs might carry and transfer some miRs to participate in vascular leakage and lung injury after sepsis (Zhou et al., 2014).

To test this hypothesis, we used the cecal ligation and puncture (CLP) induced sepsis rats and lipopolysaccharide (LPS) stimulated vascular endothelial cells (VEC), and the role and the underlying mechanisms of MVs in vascular leakage and lung injury after sepsis were investigated in the present study.

## Materials and Methods

### Ethical Approval of the Study Protocol

All animal experiments were performed in accordance with the principles of the Guide for the Care and Use of Laboratory Animals published by the United States National Institutes of Health (NIH Publications, eighth edition, 2011) and was approved by the Research Council and Animal Care and Use Committee of the Research Institute of Surgery (Daping Hospital, Army Medical University, Chongqing, China, No. DHEC-2012-069).

### Patients Information

The current study included 14 sepsis patients and 15 healthy subjects. Between January and December 2016, 14 sepsis patients were recruited from ICU of Daping Hospital, Army Medical University by reference of the criteria of Surviving Sepsis Campaign (2016 Version), and 4 patients were excluded because of age (out of range of 20−60). 15 healthy volunteers were recruited from the State Key Laboratory of Trauma, Burns and Combined Injury. Demographic characteristics of 10 sepsis patients and 15 healthy volunteers are shown in Supplementary Table S1. The study protocol was approved by the Ethics Committee of the Research Institute of Surgery, and the study was registered in Clinical Trial Registry (ChiCTR-OOC-16008352). Peripheral venous blood samples (30 mL) were collected from every patient and healthy volunteer, and microvesicles were subsequently isolated and then stored in −80°C.

### Animals Preparation and Sepsis Model

Adult male and female Sprague-Dawley (SD) rats (200−220 g) which were obtained from the Animal Center of Army Medical Center, Army Medical University (Third Military Medical University) were used in the current study. The sepsis model of rats was induced by cecal ligation and puncture (CLP) procedure with aseptic methods as described previously (Liu et al., 2016a). Briefly, rats were anesthetized by pentobarbital sodium (30 mg/kg IP), then the cecum was fully exposed and ligated 0.7 cm from the end. Afterward the ligated cecum was punctured by a triangular pyramid, and the puncture incision was about 0.1 cm × 0.1 cm. Then the cecum was put back into the enterocoelia, and the abdomen was closed by interrupted suture, and subsequently 5 mL of normal saline was injected intraperitoneally. After the operation the rats were returned to the cages and allowed for food and water *ad libitum*.

### Cell Preparation

Vascular endothelial cells were obtained from the pulmonary vein of SD rats as described previously (Liu et al., 2016a,b). Briefly, rats were anesthetized and sterilized with iodine, then rats received thoracotomy by sterile instruments. The pulmonary veins were separated from hilus pulmonis after the heart was cut off. After washed with sterile PBS for 5 times, the veins were sheared to 1 mm × 1 mm pieces, and the inner surface of the veins wad attached on the bottom of the culture flask with 5 mL ECM (Scicell, America; 5% fetal bovine serum, 1% antibiotics) medium. 3 days later, the pieces were removed from the flask, and the cells crawling on at the bottom of the culture flask were VECs, and the 3−5 passage of VECs were used in the present study. Platelets were obtained from the blood of SD rats as follows: abdominal aorta blood was collected in EDTA blood tubes (BD, America). Afterward the blood was centrifuged at 200 × *g* for 15 min (room temperature) to remove blood cells, and the supernatant was platelet rich plasma. Then platelet rich plasma was centrifuged at 1,200 × *g* for 15 min (room temperature), and the sediment was purified platelets. After washed twice by D-hanks solution, the sediment was suspended in 5 mL Dulbecco-modified Eagle medium-F12 (Hyclone, Logan, UT, United States) supplemented with 20% fetal bovine serum and 1% antibiotics (Mairhofer et al., 2002). Leukocytes were obtained according to the instruction of Leucocyte separation kit (TBD, China) and were then cultured in 5 mL DMEM-F12 medium.

### MV Harvest and Characterization

Microvesicles were harvested from blood and cultured cell supernatant according to the experiments. Blood samples were collected in BD-EDTA tubes, and cell media supernatant samples were collected (after stimulated by 1 μg/mL LPS (Sigma, America) for 24 h in basal medium without serum) in sterile centrifuge tubes. The samples were centrifuged at 500 × *g*, 15 min at 4°C to remove the residual cells. Then the supernatant was carefully collected and centrifuged at 2000 × *g*, 20 min at 4°C to remove cell debris. And the supernatant was centrifuged at 20,000 × *g*, 70 min at 4°C to pellet MVs. After washed by sterile PBS, the MVs were finally centrifuged at 20,000 × *g*, 70 min at 4°C, the pellet was suspended in PBS and stored in −80°C until use (Mastronardi et al., 2011; Matthay, 2017).

Microvesicles were analyzed by transmission electron microscopy, dynamic light scattering and flow cytometry, respectively. For transmission electron microscopy (TEM) analysis, the MV pellet was fixed with 2.5% glutaraldehyde in PBS at 4°C for 24 h. After rinsing twice with 0.1 M PBS, samples were then postfixed in 1% OsO_4_ at room temperature for 70 min. After rinsing 3 times with 0.1 M PBS, samples were dehydrated by a series of graded ethanol. Finally, the samples were embedded in TAAB 812 and 100 nm sections were prepared on grids. MV was analyzed by JEM 1400 (JEOL Instruments) transmission electron microscopy.

Dynamic light scattering analysis was performed by Zetasizer Nano ZS (Malvern Instruments) at room temperature with 633 nm He-Ne laser, automatic attenuator. Each sample was measured 3 times at least.

For flow cytometry (FCM) analysis, MVs were applied on high sensitivity imaging flow cytometry Amnis ImageStream MK II (IS^*X*^) (Amnis/Millipore, Seattle, United States), detected by X40 magnification, high sensitivity using INSPIRE software. After washed and suspended by 100 μl PBS, MVs were subsequently stained with CD31 (FITC, 1:100), CD61 (PE, 1:100), CD45 (V450, 1:100), and Annexin V (APC, 1:50) for 25 min at room temperature in the dark according to the experiment design, and all antibodies for FCM were purchased from BD, America. Annexin V-binding buffer (BD, America) was used when MVs were stained with Annexin V. Different sizes of standard beads (0.2, 0.5, and 0.8 μm, Bangs laboratories, America) were used to set gate for MVs. Isotype of matched antibodies were used as controls.

### Preparation of PKH-26 Labeled EMVs

Microvesicles were labeled by red fluorescent dye PKH-26 (Sigma, America) according to the instruction. Briefly, after washed by PBS, MVs were incubated with PKH-26 by 1:1000 at 37°C for 30 min, then MVs were centrifuged at 20,000 × *g*, 70 min to remove the remaining dye. After washed by PBS, PKH-26 labeled MVs were added into rats or VECs according to the experiment.

### Measurement of Vascular Leakage of Lung, Kidney, and Intestine

Rats were anesthetized, then FITC-BSA (9 mg/kg) was injected to rats from jugular vein. 2 h later, 20 ml PBS was used to flush vessels in each tissue (lung, kidney or intestine). In detail, 20 ml PBS was slowly injected from the jugular vein to flush the vessels in lung; and 20 ml PBS was slowly injected from the renal artery to flush the vessels in kidney after laparotomy; and an equal volume of PBS was slowly injected from the superior mesenteric artery to flush the vessels in intestine after laparotomy. Afterward rats were sacrificed by euthanasia, then left lung, left kidney and jejunum (about 6 cm) were separated. After washed by PBS, tissues were weighed up and cut into pieces, followed by complete homogenate in 5 mL PBS, then the fluorescence intensity of FITC-BSA was measured by F-4500 fluorescence spectrophotometer (Hitachi Corporation, Japan). We used the term “BSA leakage” to represent the extent of vascular leakage, and BSA leakage of each tissue was calculated by fluorescence intensity (A.U.) / tissue weight (g) (Zhang et al., 2015).

### Bronchoalveolar Lavage (BAL) Collection and the Measurement of Protein and Cell Counts in BAL

Bronchoalveolar Lavage was performed by instilling 2 mL of cold PBS into the trachea. The BAL fluid was obtained by centrifugation at 500 × *g* for 20 min (4°C), and the supernatant was used to measure protein concentration with Pierce BCA protein assay kit (Thermo Fisher Scientific, America). Then the sediment was suspended by 1 mL PBS, and the BAL cell counts were performed by cell counting chamber (Gotzfried et al., 2018; Izzotti et al., 2018).

### Transmembrane Electrical Resistance (TER) and BSA Leakage of VECs

Transmembrane Electrical Resistance and BSA leakage of VECs were measured as previously described (Zhang et al., 2015; Liu et al., 2016a,b). Briefly, VECs were seeded on upper inserts (100,000 cells per well) of a 6 cell Transwell cultureplate, (polycarbonate, 0.4 μm, Corning). Different stimuli (LPS or MVs) were treated with VECs according to the experiment design after VECs were full confluence, and TER of VECs was assessed by Voltohmmetre (World Precision Inc, America) every 30 min. BSA leakage of VECs was measured after TER analysis, FITC-BSA (10 μg/ml) was added into upper inserts of the Transwell, and 200 μl of the medium of the lower chamber at 10, 20, 30, 40, 50, and 60 min was collected for the measurement of fluorescence intensity, and an equal volume of fresh medium was added into the lower chamber after medium collection. The formula of the BSA leakage was as follows: BSA leakage (%) = (A10+A20+A30+A40+A50+A60) / total fluorescence intensity, and Ax represented the fluorescence intensity at x min.

### HE Staining and Immunofluorescence (FITC-BSA Leakage) of Lung

Rats were anesthetized and the lung was flushed with PBS. Then left lung was formaldehyde-fixed followed by paraffin-embedded for hematoxylin and eosin (HE) staining. The slides were observed by Leica microscope (Germany). For immunofluorescence of lung, rats were anesthetized and injected with FITC-BSA (9 mg/kg) for 2 h. After flushed with PBS, the left lung was formaldehyde-fixed followed embedded in Tissue-Tek O.C.T. Compound (Sakura, America). Afterward frozen sections were performed and the slides were stained with DAPI (Thermo, America), then the leakage of FITC-BSA in lung was observed by Laser confocal microscope (Zhou et al., 2014).

### FITC-BSA Leakage of Mesenteric Microvessels

Rats were anesthetized and received laparotomy, then the ileocecal portion of the mesentery was exposed and placed in a transparent plastic stage. The surface of mesentery was moisturized with 37°C saline throughout the whole procedure to keep it warm and moist. Then rats were injected with FITC-BSA (20 mg/kg) intravenously. 10 min after basal observation, fluorescence intensity of FITC-BSA in mesenteric microvessels was measured at 0, 5, and 10 min by virtue of inverted intravital microscopy (HAMAMATSU, Japan) using Image-Pro Plus 5.0 software (Li et al., 2014).

### Immunofluorescence (IF) of VECs

Immunofluorescence staining of VECs were measured as previously described with some adjustment (Zhang et al., 2015). Briefly, VECs were seeded on Confocal Petri dishes (Corning, America) and subjected with appropriate treatment at 70% confluence. Then VECs were fixed in 4% formaldehyde for 15 min (room temperature), and permeabilized with 0.1% Triton-100 for 3 min (room temperature), blocked with 5% BSA (Sigma, America), then probed with ZO-1 antibody (Thermo, America) and FITC conjugated secondary antibody (Thermo Fisher Scientific, America). The dishes were analyzed by confocal scanning microscope (Leica, Germany).

### RNA Extraction, RT-qPCR

MiRNA was extracted from microvesicles or VECs using the miRCute RNA isolation kit (TIANGEN) following the manufacturer’s instructions. The isolated RNA was reverse transcribed using the All-in-One^TM^ miRNA RT Detection kit (Genecopoeia, America). RT-PCR was performed using the All-in-One^TM^ miRNA qPCR detection kit (Genecopoeia, America) on a C1000^TM^ Thermal Cycler Real-Time PCR system from Applied Biosystems (Bio-Rad, United States). Fold induction was calculated using the Ct method as follows: ΔΔCt = (Ct target miRNA - Ct U6), and the final values were determined using 2^–ΔΔ*Ct*^ (Zhang et al., 2010).

### Transfection of VECs and Harvest of Modified EMVs

To generate modified EMVs, VECs were transfected with 2 nM miR-23b inhibitor, miR-23b mimic or miR-23b vehicle (synthesized by Genecopoeia) with lipofectamine 2000 (Invitrogen) when VECs were 70% confluence, respectively. 12 h after transfection, the medium was changed to serum-free medium, and transfected VECs were stimulated with or without LPS for another 24 h, then modified EMVs were harvested. MiR-23b mimic labeled with FAM (green) at the 3’ end was synthesized by Genecopoeia, and the sequence of miR-23b mimic and inhibitor were as follows:

miR-23b mimic;
5′-AUCACAUUGCCAGGGAUUACC-3′ Sense strand;
5′-GGUAAUCCCUGGCAAUGUGAU-3′ Antisense strand;
miR-23b inhibitor;
5′-GGUAAUCCCUGGCAAUGUGAU-3′.

### Dual Luciferase Reporter Assay for the Binding of ZO-1

Luciferase assay was performed to determine the effect of miR-23b on mRNA expression of ZO-1 and the 3’ untranslated region (UTR) of ZO-1 (Liu et al., 2016b). Briefly, VECs were seeded in 6-well plates and transfected with miR-23b overexpressed plasmid. After 24 h, luciferase reporter vector of ZO-1 (psiCHECK vector), mutant luciferase vector and control vector were transfected by Lipofectamine 2000. VECs were lysed after 24 h, and lysates were measured by dual luciferase reporter system (Promega, San Luis Obispo, CA, United States) on a 20/20-nl luminometer (Promega). Firefly luciferase activity of every sample was normalized to that of Renilla luciferase activity to correct the variations in transfection efficiency between samples.

### Western Blot

Vascular endothelial cells were lysed, and cell-protein extracts were separated by SDS−PAGE and transferred to PVDF membrane, then probed with appropriate antibodies and analyzed by Odyssey Clx (LI-COR, America). ZO-1, VE-cadherin were purchased from Thermo, and Occludin and β-actin were purchased from CST (America) (Zhang et al., 2015).

### Statistical Analysis

Data of animal studies were repeated at least 8 independent experiments. Data of cell studies were repeated at least 6 independent experiments (data of western blot was repeated for 3 times), and data from one representative experiment were shown. Data from miRNA expression were presented as mean ± SEM, and other data were presented as mean ± SD. Statistical analysis was performed by SPSS 19.0. Differences among or between groups with *n* ≥ 6 were analyzed by one-way ANOVA or independent *T*-test. The significance of data from western blot was determined by one-way Kruskal-Wallis of variance. Values of *p* < 0.05 were considered significant.

## Results

### Different Types of MV Were All Increased After Sepsis, and Led to Vascular Leakage of Vital Organ and Lung Injury

In order to investigate the role of MVs in pulmonary vascular leakage and lung injury after sepsis, MVs were purified from the blood of normal (ctl-MV) and CLP rats (CLP-MV) by ultracentrifugation, and the concentration of MVs, pulmonary vascular leakage and lung injury were observed. MVs from CLP rats were injected into rats intravenously, and the effect of MVs on pulmonary vascular leakage and lung injury were studied.

**FIGURE 1:**
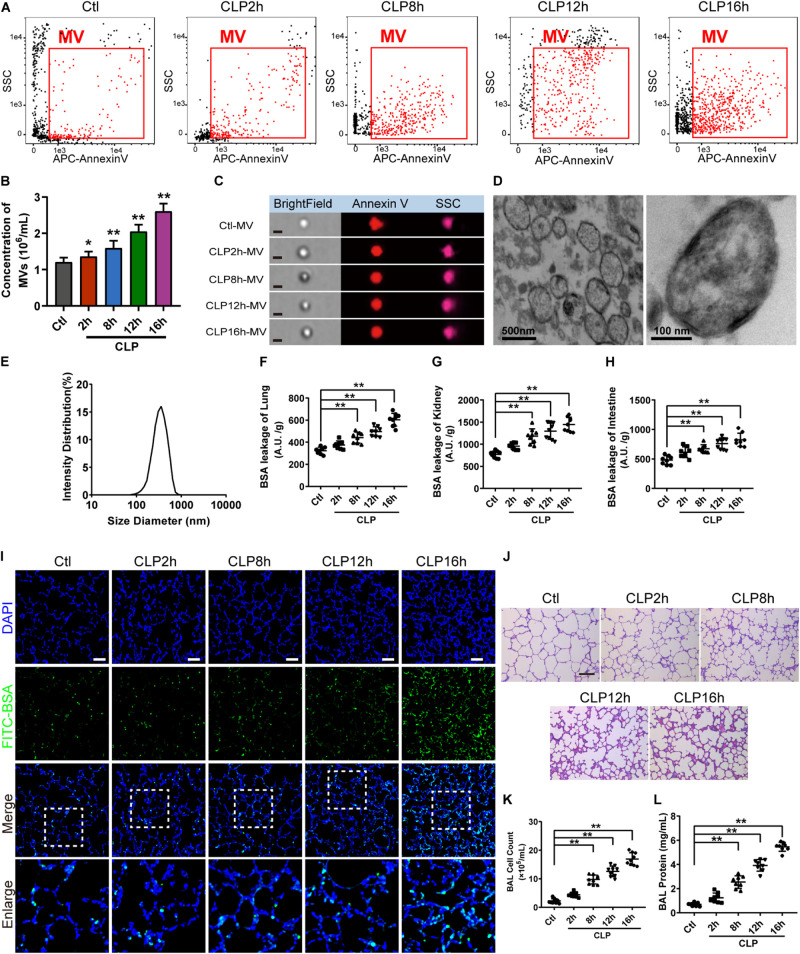
MV concentration, vascular leakage and lung injury increased in sepsis rats. **(A)** Flowcytometry analysis of MVs. The detection gate (0.1–1 μm) was set on SSC and APC channel by standard beads. **(B)** MV concentration measured by flowcytometry. **(C)** Representative flowcytometry microphotographs of MVs from different groups. The scale bar represents 1 μm. **(D)** Representative transmission electron microscope microphotographs of MVs harvested from CLP (cecal ligation and puncture) rats. **(E)** Dynamic light scattering analysis of the diameter of MVs. **(F–H)** Vascular BSA leakage of lung, kidney and intestine in CLP rats (*n* = 8), measured by the detection of the fluorescence intensity of FITC-BSA accumulation in 2 h in tissue homogenate. **(I)** BSA leakage of lung in CLP rats, determined by the appearance of intravenously injected FITC-BSA *in vivo*, representative photograph was shown. The scale bar represents 50 μm. **(J)** Lung injury in CLP rats accessed by HE staining, representative photograph was shown. The scale bar represents 100 μm. **(K)** Counts of total cells in BAL from CLP rats (*n* = 8). **(L)** Protein concentration in BAL from CLP rats (*n* = 8). Ctl, normal control; clt-MV, MVs from normal rats; CLP-MV, MVs from sepsis rats; *P < 0.05 and **P < 0.01.

For flow cytometry results showed that MVs carried externalized phosphatidylserine (Figure 1A), which was considered to be the marker of MVs in most cases, and the majority of MVs was 200−1000 nm. Furthermore, imaging flow cytometry showed the exact image of MVs, which exhibited spherical structure (Figure 1C). The concentration of MVs from the normal rats (ctl-MV) was 1.19 × 10^6^/mL, and they were increased to 1.26, 1.53, 1.82, 2.11 × 10^6^/mL at 2, 8, 12, and 16 h after sepsis, respectively (Figure 1B), and the increment rate was 6, 29, 53, and 77%. MVs exhibited spherical morphology with typical bimolecular membrane structure and the size was from 100 to 1000 nm in electron microscopy (Figure 1D). Verification by dynamic light scattering, the size of MVs was from 82.09 to 859.2 nm with a mean size of 352.98 nm (Figure 1E). The size and morphology of MVs obtained were accordant with the existing literatures (Mastronardi et al., 2011; Tkach and Thery, 2016; Martinez and Andriantsitohaina, 2017).

The vascular leakage of lung was significantly aggravated after sepsis (Figure 1F), along with the change of vascular leakage of kidney (Figure 1G) and intestine (Figure 1H). Immunofluorescence of lung showed that a small amount of FITC-BSA could be found in pulmonary interstitium at 2 h, while the leakage of FITC-BSA was significantly enhanced at 8 h after sepsis, which appeared a time dependent manner after sepsis (Figure 1I). Meanwhile, lung injury was also aggravated in a time dependent manner, which was characterized by tissue edema, leukocyte and erythrocyte infiltration (Figure 1J). The cell counts and protein in bronchoalveolar lavage fluid (BAL) were also significantly increased after sepsis (Figures 1K,L). Considering that the vascular leakage had the highest increase rate at 16 h after sepsis, we chose the 16 h time point as the observation time point for CLP model in the following experiments.

**FIGURE 2:**
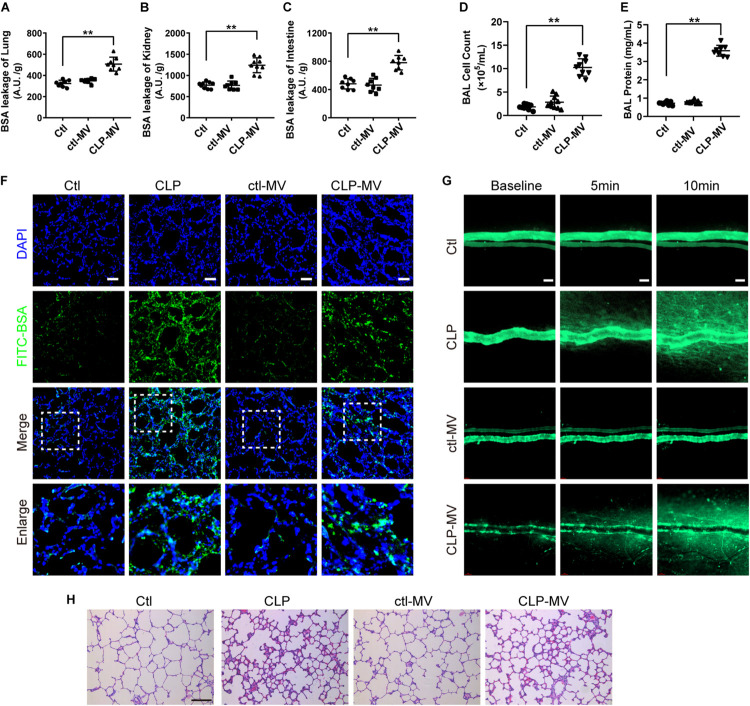
MVs obtained from sepsis rats induced pulmonary vascular leakage and lung injury. **(A–C)** Vascular BSA leakage of lung, kidney and intestine in rats injected with MVs (*n* = 8), measured by the detection of the fluorescence intensity of FITC-BSA accumulation in 2 h in tissue homogenate. **(D,E)** Cell counts and protein concentration in BAL from rats injected with MVs (*n* = 8). **(F)** BSA leakage of lung in rats injected with MVs, determined by the appearance of intravenously injected FITC-BSA *in vivo*, representative photograph was shown. The scale bar represents 50 μm. **(G)** BSA leakage of mesentery microvessel in rats injected with of MVs, dynamically measured by inverted intravital microscopy *in vivo*. The scale bar represents 50 μm. **(H)** Lung injury in rats injected with MVs accessed by HE staining, representative photograph was shown. The scale bar represents 100 μm. Ctl, normal control; clt-MV, MVs from normal rats; CLP-MV, MVs from sepsis rats; **P < 0.01.

To further identify the role of MVs in pulmonary vascular leakage and lung injury after sepsis, MVs from normal and sepsis rats were injected into normal rats (1 × 10^7^ MVs, 2 times, every 8 h), the pulmonary vascular leakage and lung injury were measured at 16 h after the first injection. The results showed that pulmonary vascular leakage was significantly increased after injection of MVs from CLP rats, along with the vascular leakage in kidney and intestine (Figures 2A–C), while MVs from normal rats had no effect on vascular leakage. Meanwhile, MVs from CLP rats led to obvious leakage of FITC-BSA in lung and mesenteric microvessels (Figures 2F,G), induced tissue edema, leukocyte and erythrocyte infiltration in lung (Figure 2H), and increased the cell counts and protein in BAL (Figures 2D,E). These results indicate that MVs play an important role in vascular leakage of vital organ and lung injury after sepsis.

**FIGURE 3:**
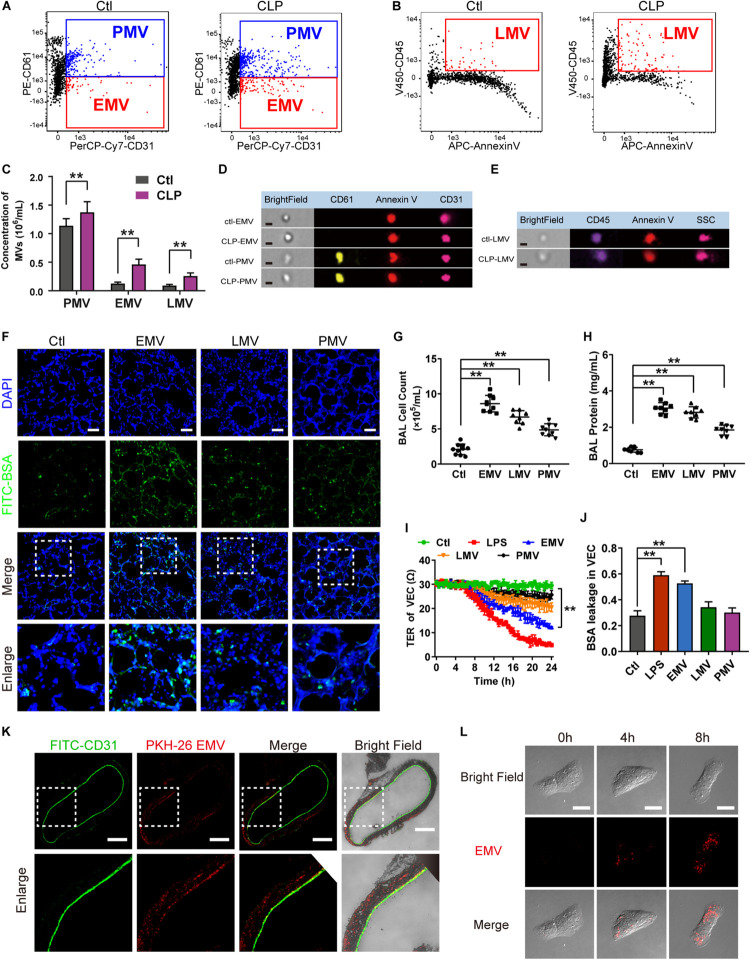
EMVs led to pulmonary vascular leakage and lung injury after sepsis. **(A–C)** Flowcytometry analysis of PMVs, EMVs, and LMVs from CLP rats. CD61+/CD31+ represents PMVs, and CD61-/CD31+ represents EMVs, and CD45+/Annexin V+ represents LMVs. **(D,E)** Representative flowcytometry microphotographs of different MVs. The scale bar represents 1 μm. **(F)** BSA leakage of lung in rats injected with different MVs, determined by the appearance of intravenously injected FITC-BSA *in vivo*, representative photograph was shown. The scale bar represents 50 μm. **(G,H)** Cell counts and protein concentration in BAL from rats injected with different MVs (*n* = 8). **(I)** TER (transmembrane electrical resistance) of VEC monolayers incubated with different MVs. **(J)** BSA leakage of VECs incubated with different MVs (*n* = 6). **(K)** Endocytosis of PKH-26 labeled EMV (red) in pulmonary vein, representative photograph was shown. The scale bar represents 200 μm. **(L)** Endocytosis of PKH-26 labeled EMV (red) in VECs, representative photograph was shown. Ctl, normal control. The scale bar represents 20 μm. **P < 0.01.

There are several types of MV in blood, including endothelial MVs (EMV), platelet MVs (PMV), leukocyte MVs (LMV), etc. To further investigate which type of MV plays the leading role in the regulation of vascular leakage, MVs from CLP rats were applied on FCM to measure the exact profile of different MVs. The concentration of EMVs, PMVs and LMVs (Figures 3D,E) were 1.07 × 10^5^/mL, 9.05 × 10^5^/mL, 9.66 × 10^4^/mL in control group, respectively (Figures 3A,B). In sepsis rats, the concentration of EMVs, PMVs and LMVs increased to 4.27 × 10^5^/mL, 1.32 × 10^6^/mL, and 2.17 × 10^5^/mL, respectively (Figure 3C). The increment rates were 3.99, 1.46, and 2.25 fold at 16 h after sepsis, and the concentration of EMVs was increased most after sepsis.

Furthermore, different types of MVs were harvested from LPS stimulated VECs, platelets and leukocytes, which were also injected into normal rats (1 × 10^7^ MVs, 2 times, every 8 h), the same results were obtained as in MVs harvested from CLP rats, which showed pulmonary vascular leakage was significantly aggravated in EMV group after injection, while pulmonary vascular leakage was slightly increased in PMV and LMV group (Figure 3F). EMVs and LMVs induced apparent increment in cell counts and protein infiltration in BAL (Figures 3G,H), while PMVs injection led to minor increment on cell counts and protein infiltration.

Subsequently, EMVs, PMVs and LMVs were applied to stimulate VECs with a dose of 1 × 10^6^. The results showed that EMVs reduced the transmembrane electrical resistance (TER) of VEC significantly, while LMV and PMV only slightly reduced the TER of VEC (Figure 3I). Meanwhile, the BSA leakage of VECs was significantly increased after incubation of EMVs, while LMVs and PMVs had little effect on the BSA leakage of VECs (Figure 3J). These results indicate that EMVs are the most pivotal type of MVs which play the leading role in pulmonary vascular leakage and lung injury after sepsis.

### EMVs Induced Pulmonary Vascular Leakage and Lung Injury by Transferring miR-23b

To investigate how EMVs interact with VECs, red fluorescent dye PKH-26 labeled EMVs were injected into normal rats via femoral vein with a dose of 1 × 10^7^, 8 h after injection the pulmonary vein was isolated and observed by the confocal fluorescence microscopy. Immunofluorescence showed that PKH-26-labeled EMVs were taken up by the vascular endothelium (green fluorescence showed CD31+) (Figure 3K). Interestingly, PKH-26 labeled EMVs could be also found in the sub-endothelial and media of the pulmonary vein, which indicated that EMVs could penetrate the endothelial barrier to the depth of blood vessels, and played an important role in sepsis. Furthermore, PKH-26-labeled EMVs were incubated with VECs, and results showed that EMVs were incorporated into VECs in a time dependent manner (Figure 3L), confirming both vascular endothelium and VEC can uptake EMVs.

**FIGURE 4:**
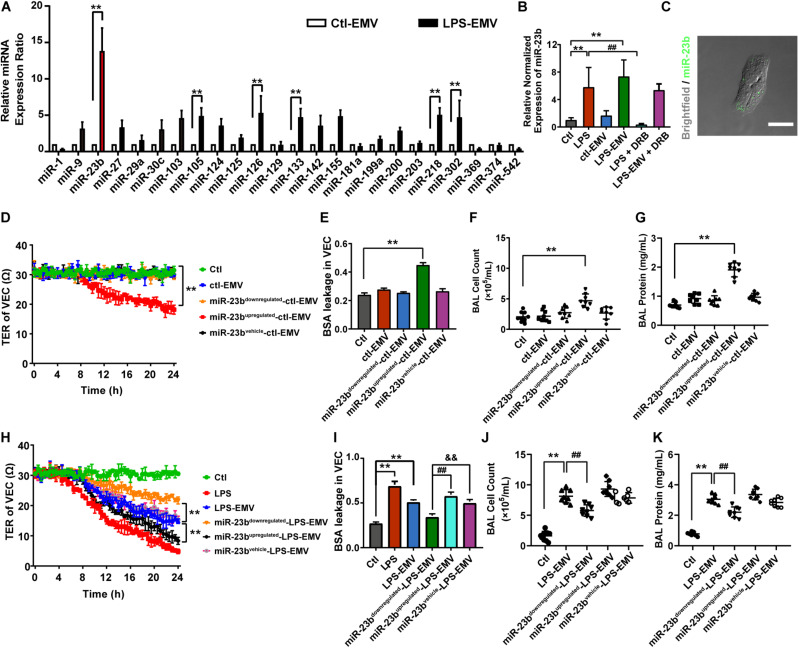
The expression profile of miRNA in EMVs and the effects of EMVs with miR-23b inhibition and overexpression on vascular leakage. **(A)** Comparison of miRNA expression profile in EMVs obtained from VECs treated with (LPS-EMVs) or without (ctl-EMVs) LPS (100 ng/mL), measured by quantitative RT-PCR. **(B)** Quantitative RT-PCR analysis of miR-23b in VECs incubated with LPS-EMVs in the presence of DRB (5,6-Dichlorobenzimidazole1-β-D-ribofuranoside). DRB is RNA polymerase II inhibitor, which can interfere the endogenous synthesis of RNA. **(C)** Representative microphotograph of the internalization of EMVs carrying FAM-miR-23b into VECs. The scale bar represents 20 μm. **(D)** TER of VEC monolayers incubated with different types of ctl-EMVs. **(E)** BSA leakage of VECs incubated with different types of ctl-EMVs (*n* = 6). **(F,G)** Cell counts and protein concentration in BAL from rats injected with different types of ctl-EMVs (*n* = 8). **(H)** TER of VEC monolayers incubated with different types of LPS-EMVs, LPS group was the positive control (the same below). **(I)** BSA leakage of VECs incubated with different types of LPS-EMVs (*n* = 6). **(J,K)** Cell counts and protein concentration in BAL from rats injected with different types of LPS-EMVs (*n* = 8). Ctl, normal control; ctl-EMV, EMVs from normal VECs; LPS-EMV, EMVs from VECs treated with LPS; **P < 0.01, ^&&^*P* < 0.01 and ^##^*P* < 0.01.

To investigate whether EMVs induce pulmonary vascular leakage after sepsis by carrying miR, we observed the miR expression profile in EMVs harvested from LPS treated VECs (LPS-EMV) and untreated VECs (ctl-EMV) at first. 24 miRNAs which were highly related to vascular leakage and lung injury were measured. The results showed that the expression of miR-23b, miR-105, miR-126, miR-133, miR-218, and miR-302 in LPS-EMVs were significantly higher than ctl-EMVs (Figure 4A), during which the most enriched miRNA in LPS-EMVs was miR-23b, with a 13.58-fold increment rate.

To verify whether EMVs transfer miR-23b into VECs, we measured the expression of miR-23b in VECs after incubation by LPS-EMVs and ctl-EMVs. The results showed that LPS-EMVs stimulation effectively increased the expression of miR-23b in VECs while ctl-EMVs had no effect (Figure 4B). Furthermore, we transfected FAM-labeled (green fluorescence) miR-23b into VECs to generate containing green fluorescence EMVs, which were then incubated with target VECs. Microscopically, FAM-labeled miR-23b could be found in the cytoplasm of target VECs, which provided a visual proof that miR-23b was transferred into VECs by EMVs (Figure 4C). To further investigate whether the increase of miR-23b in VECs was from the EMV-carried miR, RNA polymerase II inhibitor DRB was used to interfere the endogenous synthesis of RNA in target VECs. DRB showed no influence on the increase of miR-23b after EMV stimulation, which indicated that the increase of miR-23b in target VECs was resulted from the EMV contained miR-23b transfer rather than endogenous miR-23b in target VECs (Figure 4B).

In order to investigate whether EMVs induce pulmonary vascular leakage and lung injury by transferring miR-23b, the miR-23b inhibitor and miR-23b mimic were used to prepare modified EMVs. In the present study, VECs were firstly transfected as described, and then treated with or without LPS, thus we defined the modified EMVs according to the sequence of treatment as: miR-23b^*upregulated*^-LPS-EMV, miR-23b^*downregulated*^-LPS-EMV, miR-23b^*vehicle*^-LPS-EMV, miR-23b^*upregulated*^-ctl-EMV, miR-23b^*downregulated*^-ctl-EMV and miR-23b^*vehicle*^-ctl-EMV, respectively.

Then modified EMVs treated without LPS were incubated with VECs, and miR-23b^*downregulated*^-ctl-EMVs and miR-23b^*vehicle*^-ctl-EMVs showed no effect on TER and BSA leakage of VECs, while incubation of miR-23b^*upregulated*^-ctl-EMVs led to the decrease of TER of VECs, and the increase of BSA leakage of VECs (Figures 4D,E). Meanwhile, incubation of miR-23b^*upregulated*^-ctl-EMVs induced apparent increase of cell count and protein in BAL (Figures 4F,G).

To further confirm the role of miR-23b in vascular leakage and lung injury, modified EMVs treated with LPS were applied to stimulate VECs. Compared with control group, LPS-EMVs induced significant leakage in VECs, while miR-23b^*downregulated*^-LPS-EMVs abolished the LPS-EMV induced leakage of VECs, and miR-23b^*upregulated*^-LPS-EMVs further aggravated the leakage (Figures 4H,I). Furthermore, miR-23b^*upregulated*^-LPS-EMVs aggravated the increment of cell count and protein effusion in BAL caused by LPS-EMVs (Figures 4J,K). These results indicated that the EMV transferring miR-23b induced pulmonary vascular leakage and lung injury.

### EMVs Induced Vascular Leakage and Lung Injury by Inhibiting ZO-1 Expression

In general, there are several key proteins in the regulation of vascular leakage: tight junction protein ZO-1, Occludin and adherent junction protein VE-cadherin. To investigate which protein may take part in vascular leakage and lung injury by miR-23b containing EMV, at first we predicted the potential target of miR-23b by Targetscan, and found ZO-1 mRNA might be the most feasible target of miR-23b among those targets (Data not shown).

**FIGURE 5:**
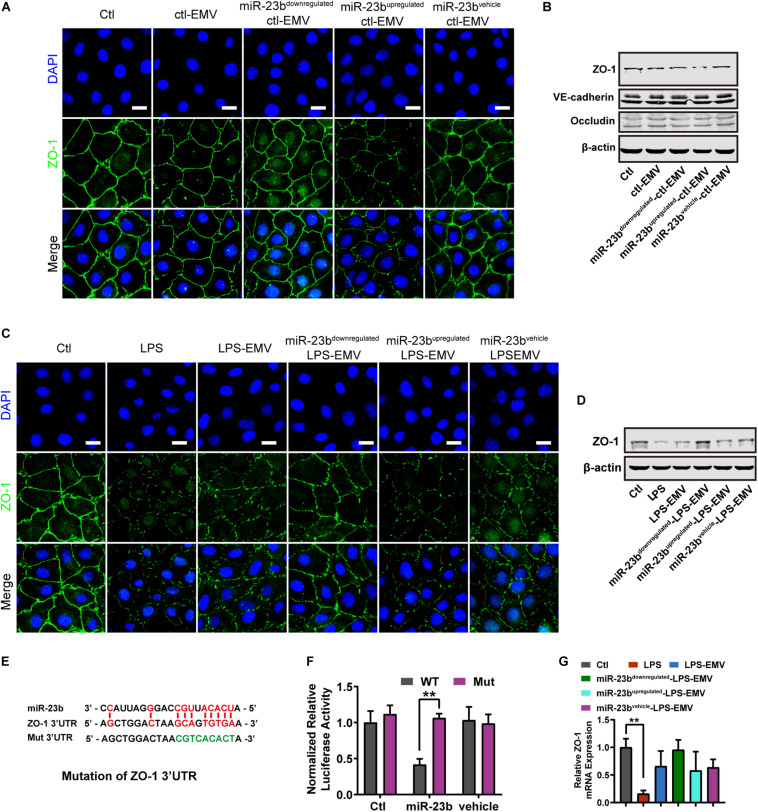
The effect of EMVs with miR-23b inhibition and overexpression on ZO-1 expression. **(A)** Immunofluorescence for ZO-1 in VECs incubated with different types of ctl-EMVs. The scale bar represents 20 μm. **(B)** Western blot analysis of ZO-1 in VECs incubated with different types of ctl-EMVs (*n* = 3). **(C)** Immunofluorescence for ZO-1 in VECs incubated with different types of LPS-EMVs. LPS group was the positive control (The same below). The scale bar represents 20 μm. **(D)** Western blot analysis of ZO-1 in VECs incubated with different types of LPS-EMVs (*n* = 3). **(E)** Schematic showing miR-23b predicted binding sites in ZO-1 3’-UTR and mutant ZO-1 3’-UTR sequence (Mut). Mut sequence of ZO-1 3’-UTR was introduced by replacing the wild type binding sequence (GCAGTGTGA) with a mutant sequence (CCTTTACAT, in green). **(F)** Effects of miR-23b on the 3’-UTR of ZO-1 and the mutant of ZO-1, measured by Dual-Luciferase System. **(G)** Quantitative RT-PCR analysis of ZO-1 mRNA in VECs incubated with different types of LPS-EMVs. Ctl, normal control; ctl-EMV, EMVs from normal VECs; LPS-EMV, EMVs from VECs treated with LPS; **P < 0.01, ^##^*P* < 0.01, and &&*P* < 0.01.

Further experiment found that ctl-EMVs and miR-23b^*downregulated*^-ctl-EMVs showed no influence on the expression of ZO-1, and miR-23b^*upregulated*^-ctl-EMVs significantly inhibited the expression of ZO-1 (Figure 5A) and impaired the integrity of ZO-1 barrier (Figure 5B). Meanwhile, miR-23b^*upregulated*^-ctl-EMVs had no effect on the expression of Occludin and VE-cadherin (Figure 5B), which indicated that the effect of miR-23b containing EMVs was related to ZO-1 inhibition.

Furthermore, LPS-EMVs decreased the ZO-1 expression, and miR-23b^*upregulated*^-LPS-EMVs aggravated the decrease of ZO-1 expression induced by LPS-EMVs, while miR-23b^*downregulated*^-LPS-EMVs attenuated the LPS-EMVs induced increase of ZO-1 expression (Figure 5C). Identically, miR-23b^*upregulated*^-LPS-EMVs caused more harm to ZO-1 barrier, and miR-23b^*downregulated*^-LPS-EMVs could attenuate the LPS-EMVs induced endothelial barrier damage (Figure 5D). Dual luciferase reporter assay verified that miR-23b could act on the 3’-UTR of ZO-1 mRNA (Figures 5E,F). Interestingly, LPS-EMVs had little effect on the expression of ZO-1 mRNA (Figure 5G), which suggested that miR-23b in EMVs regulated the expression by affecting the 3’-UTR of ZO-1 without degradation of the ZO-1 mRNA.

### MVs From Sepsis Patients Induced Pulmonary Vascular Leakage and Lung Injury in Rats

**FIGURE 6:**
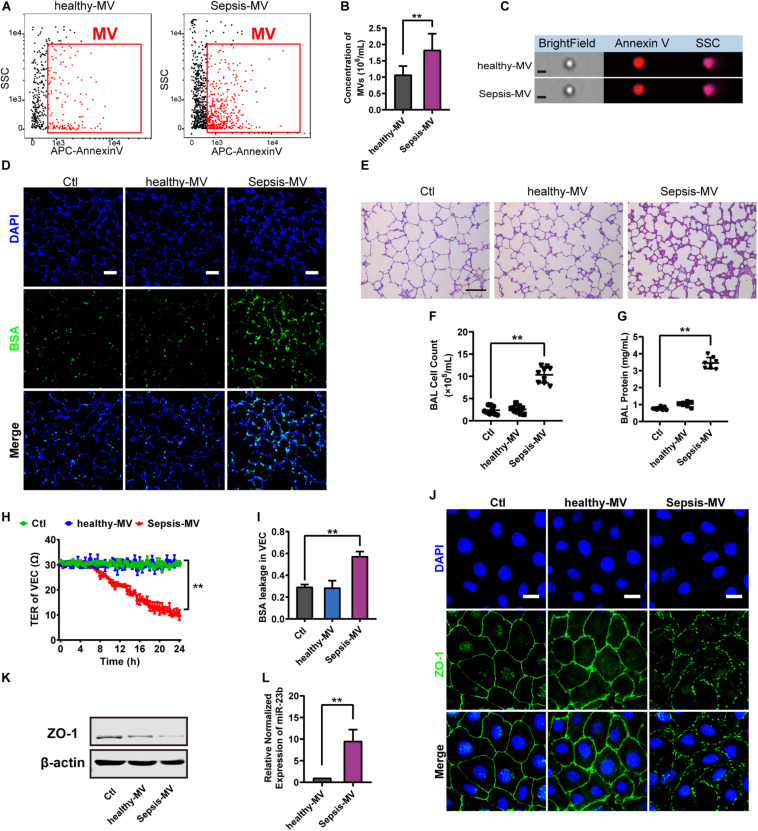
MVs harvested from sepsis patients induced pulmonary vascular leakage and lung injury. **(A,B)** Flowcytometry analysis of MVs from heathy people (healthy-MVs) and sepsis patients (sepsis-MVs). **(C)** Representative flowcytometry microphotographs of healthy-MVs and sepsis-MVs. The scale bar represents 1 μm. **(D)** BSA leakage of lung in rats injected with healthy-MVs or sepsis-MVs, determined by the appearance of intravenously injected FITC-BSA *in vivo*, representative photograph was shown. The scale bar represents 50 μm. **(E)** Lung injury in rats injected with healthy-MVs or sepsis-MVs, accessed by HE staining, representative photograph was shown. The scale bar represents 100 μm. **(F,G)** Cell counts and protein concentration in BAL from rats injected with different MVs (*n* = 8). **(H)** TER (transmembrane electrical resistance) of VEC monolayers incubated with different MVs. **(I)** BSA leakage of VECs incubated with different MVs (*n* = 6). **(I)** Immunofluorescence for ZO-1 in VECs incubated with different MVs. The scale bar represents 20 μm. **(K)** Western blot analysis of ZO-1 in VECs incubated with different MVs (*n* = 3). **(L)** Quantitative RT-PCR analysis of miR-23b in VECs incubated with different MVs. Ctl, normal control; ** *P* < 0.01.

To further verify the role of MVs on pulmonary vascular leakage and lung injury after sepsis, we collected MVs from sepsis patients to stimulate normal rats. In this experiment, 10 patients and 15 healthy adults were recruited for MV collecting (Supplementary Table S1). The concentration of circulating MVs in sepsis patients (sepsis-MV) was significantly higher than that in healthy adults (healthy-MVs) (Figures 6A–C).

Then MVs from sepsis patients were applied to normal rats, and data showed that MVs from sepsis patients induced apparent pulmonary vascular leakage in rats while MVs from healthy adults had no effect (Figure 6D). Meanwhile, MVs from sepsis patients led to apparent lung injury and protein effusion in BAL (Figures 6E–G). Furthermore, MVs from sepsis patients led to apparent leakage in VECs (Figures 6H,I). As compared with MVs from healthy adults, MVs from sepsis patients reduced the expression of ZO-1 and impaired the integrity of ZO-1 (Figures 6J,K). Meanwhile, the expression of miR-23b in MVs from sepsis patients was significantly higher than that in MVs from healthy adults (Figure 6L). These results further confirmed that miR-23b-containing MVs in sepsis patients played an essential role in pulmonary vascular leakage and lung injury.

### Inhibition of EMV Generation Ameliorates Pulmonary Vascular Leakage and Lung Injury After Sepsis

**FIGURE 7:**
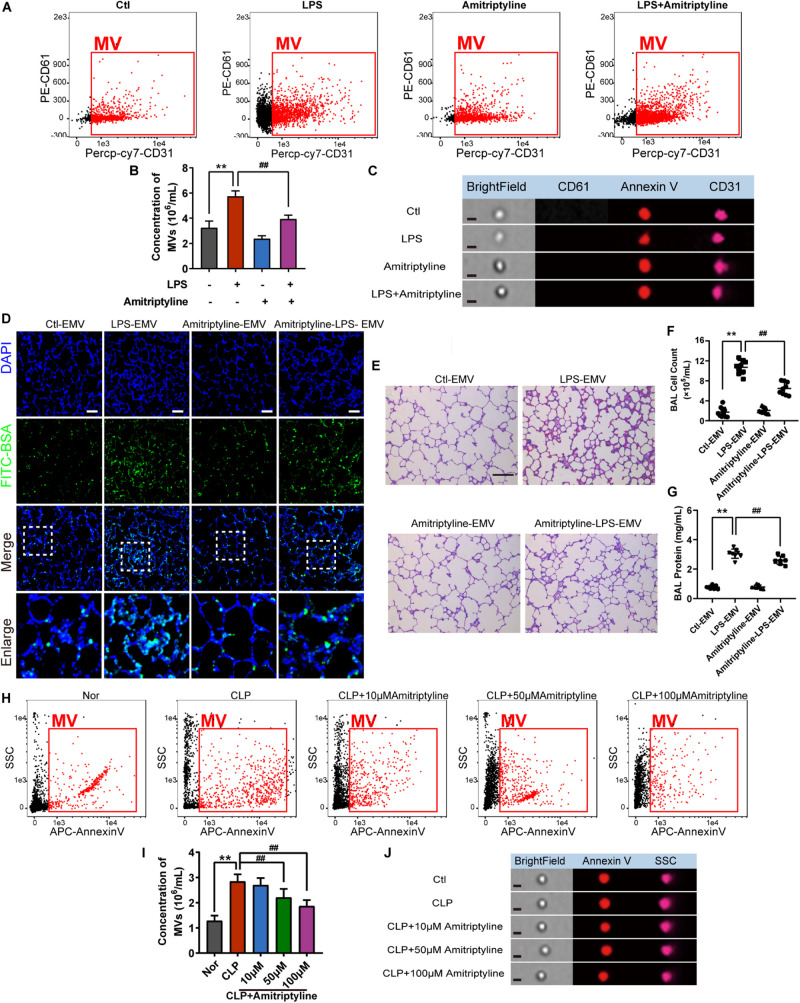
Amitriptyline inhibited the generation of EMVS and attenuated the effect of EMVs on pulmonary vascular leakage. **(A,B)** Flowcytometry analysis of EMVs from VECs treated with/without LPS or amitriptyline. **(C)** Representative flowcytometry microphotographs of different EMVs. The scale bar represents 1 μm. **(D)** BSA leakage of lung in rats injected with different EMVs, determined by the appearance of intravenously injected FITC-BSA *in vivo*, representative photograph was shown. The scale bar represents 50 μm. **(E)** Lung injury in rats injected with different EMVs, accessed by HE staining, representative photograph was shown. The scale bar represents 100 μm. **(F,G)** Cell counts and protein concentration in BAL from rats injected with different EMVs (*n* = 8). **(H,I)** Flowcytometry analysis of MVs from CLP rats treated with different doses of amitriptyline. **(J)** Representative flowcytometry microphotographs of different MVs. The scale bar represents 1 μm. Ctl, normal control; **P < 0.01 and ^##^*P* < 0.01.

As the accumulation of EMVs in blood after sepsis led to subsequent damage on vessels, we aimed to attenuate the effect of EMVs by inhibiting the generation of EMVs. 10 μM of acid SMase inhibitor amitriptyline was used to treat VEC for 24 h, and data showed that amitriptyline effectively reduced the release of EMVs (Figures 7A–C). EMVs obtained from amitriptyline and LPS treated VECs (Ami-LPS-EMV) and amitriptyline could attenuate the LPS-EMVs induced pulmonary vascular leakage (Figure 7D) and attenuate the cell count and protein infiltration in BAL (Figures 7F,G), and the lung injury (Figure 7E). *In vivo* study showed that amitriptyline could reduce EMVs production in sepsis rats in a dose-dependent manner at 50 and 100 μM (Figures 7H–J).

## Discussion

The present study showed that EMVs played an important role in pulmonary vascular leakage and lung injury after sepsis, in this process, miR-23b played a critical role. Inhibition of the release of MV including EMV with amitriptyline could significantly alleviate sepsis induced vascular leakage and lung injury. This study provides a new sight for exploration of the treatment of sepsis.

Lung injury is the leading cause of death during sepsis and the main failure organ in the development of multiple organ dysfunction after sepsis (Wu et al., 2007). Vascular leakage is one of the most important pathophysiological processes in lung injury, which is considered to be related to abundant cytokines in blood after sepsis (Hou et al., 2017). It has been proved that cytokines such as Angiopoietin2, VEGF, TGF-β and HMGB1 are important in the occurrence of vascular leakage (Hotchkiss et al., 2016; Hattori et al., 2017). For example, circulating TGF-β induced protein (TGFBIp) level was increased after sepsis, and the subsequent interaction with integrin αvβ5 could result in the increased proinflammatory responses, and promote the adhesion and migration of leukocytes across the endothelium, which eventually aggravated vascular leakage and the progress of sepsis (Bae et al., 2014). Several therapies were designed to inhibit the impact of cytokines, but the results were not satisfactory, which indicated that there were many other factors involved in the process. Recent studies showed that MVs played important roles in the occurrence and development of sepsis, including coagulation, inflammation and immunodeficiency, etc. The present study revealed that MV, especially EMVs played an important role in vascular leakage and lung injury after sepsis.

The concentration of MV in circulation is low in healthy rats, which is mainly composed of PMVs, with a proportion of up to 76.1%, while the proportion of EMVs, LMVs is low (Mastronardi et al., 2011). Previous studies have found that PMV can participate in the activation of exogenous coagulation reaction after sepsis (Delabranche et al., 2016). By expressing high-affinity binding sites for coagulation factors Va, VIII, and IXa on its surface, and carrying platelet activating factor (PAF), PMVs can react with platelets and monocyte, which can continuously activate thrombin generation thus promote coagulation process. LMVs can carry a variety of inflammatory factors from leukocytes, for example, they can transfer myeloperoxidase into endothelial cells, which will interfere with the myeloperoxidase-hydrogen peroxide-chloride system, leading to the damage of endothelial cells and inflammation aggravation (Bae et al., 2014). EMVs are mainly secreted from the damaged endothelial cells, which are related to endothelial injury, remodeling and angiogenesis (Paone et al., 2019). However, the present study revealed that the proportion of EMVs was significantly increased, from 9.0 to 20.2% in sepsis rats, which may be related to the extensive activation of vascular endothelium during sepsis. Because VECs are the first barrier for pathogenic factors when sepsis occurred (Aird, 2003). Stimulated VECs secret a large number of EMVs subsequently, which reach all parts of the body along with the circulation, leading to the extensive activation of vascular endothelium throughout the body, and inducing the aggravation of sepsis. Except for EMVs, the present study found the concentration of PMVs and LMVs were also significantly increased during sepsis. Among the three types of MVs, the present study showed that EMVs played the leading role in pulmonary vascular leakage and lung injury.

MVs could provide shelter for miRNAs to keep them from being degraded by RNase in the blood, which has been proved that miRNAs could be selectively packaged into MVs to participate in multiple diseases (Zhang et al., 2010; Zhou et al., 2014; Xu et al., 2017; Zheng et al., 2017). MiR-23b was firstly identified to be an oncogene which is related to breast cancer, glioma and kidney cancer, and it participates in the regulation of cytoskeletal reconstruction, cell invasion, metastasis and inflammatory reactions (Donadelli et al., 2014; Grossi et al., 2018). Our present study found that miR-23b was abundant in EMV after sepsis. By predicting potential target of miR-23b using Targetscan, tight junction ZO-1 was found to be the most feasible target of miR-23b, rather than tight junction Occludin nor adherent junction VE-cadherin. Tight junction ZO-1 plays an important role in maintaining the stability of the barrier of VECs, and the destruction of ZO-1 results in the leakage of vessels. Besides, there are many other miR as miR-105, miR-132 could also participate in the regulation of vascular leakage in other diseases (Zhou et al., 2014; Gu et al., 2019), but these miRs showed little change in EMVs after sepsis, which suggests that the miR-23b is selectively packaged into EMVs during the progress of sepsis, and the mechanism of selective package needs to be further studied. In addition, MVs separated from sepsis patients could also lead to pulmonary vascular leakage and lung injury in rats, and aggravate the leakage of pulmonary vascular endothelial cells, suggesting that MVs as a vehicle for intercellular information exchange can transmit biological information between species.

Amitriptyline was known to be effective for antidepressant effect in mental illness, which was found to be able to reduce acid SMase activity and the synthesis of phospholipid. Acid SMase plays a role in the synthesis of membrane lipids (Awojoodu et al., 2014; Depciuch et al., 2017), thus amitriptyline is thought able to be a non-specific drug to inhibit the secretion of MVs, and it was proved to be effective at the dose of 100 μM (Awojoodu et al., 2014). Our present study found that 50 and 100 μM amitriptyline significantly inhibited the release of EMVs during sepsis and alleviated the pulmonary vascular leakage and lung injury. This result suggests that amitriptyline may be a therapeutic candidate for the treatment of pulmonary vascular leakage induced by MVs. However, the mechanism of MV generation is complicated, which is relevant to phosphatidylserine, sphingomyelin, flippase, floppase and Ca^2+^, etc (Buzas et al., 2018; Malloci et al., 2018; Meldolesi, 2018), amitriptyline could reduce the formation of MVs to some extent, while how to effectively use amitriptyline to inhibit the secretion of MVs needs further investigation.

Nevertheless, there are some limitations in the present study. Firstly, the number of patients we recruited was a small population, whether EMVs play an important role in vascular leakage in sepsis patients needs further verification. Secondly, there are many factors such as tissue factor, MMPs etc (Krizanac-Bengez et al., 2006; Yeh et al., 2013; Lee et al., 2014), which may affect vascular barrier in addition to ZO-1, whether EMVs-induced vascular leakage is related to MMPs or TF needs to be further investigated. Thirdly, the present study was focused on the role of MVs, while exosomes may also participate in the vascular leakage because of the similar origins (Caivano et al., 2017), whether exosomes from VECs also play an important role needs further investigation.

In conclusion, our study showed that EMVs play an important role in pulmonary vascular leakage and lung injury during sepsis by transferring functional miR-23b. Antagonizing the secretion of EMVs and its miR-23b may alleviate the vascular leakage and lung injury. EMVs and miR-23b might be a potential therapeutic target for vascular leakage and organ injury in sepsis.

## Data Availability Statement

All datasets presented in this study are included in the article/ Supplementary Material.

## Ethics Statement

The studies involving human participants were reviewed and approved by the Ethics Committee of the Research Institute of Surgery (Daping Hospital, Army Medical University). The patients/participants provided their written informed consent to participate in this study. The animal study was reviewed and approved by Research Council and Animal Care and Use Committee of the Research Institute of Surgery (Daping Hospital, Army Medical University, Chongqing, China, Number DHEC-2012-069).

## Author Contributions

DZ, JZ, ZZ, and HZ performed the experiments and analyzed the data. DZ, LK, YZ, YW, and CD collected the clinical samples and approved the manuscript. LL and TL participated in the study design. All authors have read and approved the manuscript, and ensured that it is the case.

## Conflict of Interest

The authors declare that the research was conducted in the absence of any commercial or financial relationships that could be construed as a potential conflict of interest.
