# Supplementary material for: Endothelial Microvesicles Induce Pulmonary Vascular Leakage and Lung Injury During Sepsis
Source: Front Cell Dev Biol. 2020 Jul 16;8:643. doi: 10.3389/fcell.2020.00643 (PMC7379030; doi:10.3389/fcell.2020.00643)
Supplement: Supplementary file 1 [file Table_1.docx]

**TableS1.** Baseline characteristics of the sepsis patients and healthy subjects in this study.

| **Characteristics** | **Sepsis patients** | **Healthy subjects** |
| --- | --- | --- |
| Age, yrs | 39±15 | 37±10 |
| Gender, n (%) | 10 | 15 |
| Female | 4 (40%) | 4 (26.67%) |
| Male | 6 (60%) | 11 (73.33%) |
| Mean arterial Pressure, mmHg | 78.1±5.8 | 89.3±4.7 |
| White blood count, ×10^9^ | 14.8±3.3 | 6.4±1.6 |
| Heart rate/min | 86.5±9.3 | 71.4±6.2 |
| Primary illness |  |  |
| Severe pneumonia | 4 | 0 |
| Multiple injury | 3 | 0 |
| Pancreatitis | 1 | 0 |
| Nephritis | 1 | 0 |
| Pelvic fracture | 1 | 0 |
| MV concentration (1×10^6^/mL) | 1.83±0.50 | 1.07±0.27 |
